# Adjustment for baseline characteristics in randomized trials using logistic regression: sample-based model versus true model

**DOI:** 10.1186/s13063-022-07053-7

**Published:** 2023-02-13

**Authors:** Thomas Perneger, Christophe Combescure, Antoine Poncet

**Affiliations:** Division of Clinical Epidemiology, University of Geneva and Geneva University Hospitals, 6 Rue Gabrielle-Perret-Gentil, 1211 Geneva, Switzerland

**Keywords:** Randomized clinical trials, Baseline imbalance, Statistical adjustment, Over-fitting, Simulation study

## Abstract

**Background:**

Adjustment for baseline prognostic factors in randomized clinical trials is usually performed by means of sample-based regression models. Sample-based models may be incorrect due to overfitting. To assess whether overfitting is a problem in practice, we used simulated data to examine the performance of the sample-based model in comparison to a “true” adjustment model, in terms of estimation of the treatment effect.

**Methods:**

We conducted a simulation study using samples drawn from a “population” in which both the treatment effect and the effect of the potential confounder were specified. The outcome variable was binary. Using logistic regression, we compared three estimates of the treatment effect in each situation: unadjusted, adjusted for the confounder using the sample, adjusted for the confounder using the true effect. Experimental factors were sample size (from 2 × 50 to 2 × 1000), treatment effect (logit of 0, 0.5, or 1.0), confounder type (continuous or binary), and confounder effect (logit of 0, − 0.5, or − 1.0). The assessment criteria for the estimated treatment effect were bias, variance, precision (proportion of estimates within 0.1 logit units), type 1 error, and power.

**Results:**

Sample-based adjustment models yielded more biased estimates of the treatment effect than adjustment models that used the true confounder effect but had similar variance, accuracy, power, and type 1 error rates. The simulation also confirmed the conservative bias of unadjusted analyses due to the non-collapsibility of the odds ratio, the smaller variance of unadjusted estimates, and the bias of the odds ratio away from the null hypothesis in small datasets.

**Conclusions:**

Sample-based adjustment yields similar results to exact adjustment in estimating the treatment effect. Sample-based adjustment is preferable to no adjustment.

**Supplementary Information:**

The online version contains supplementary material available at 10.1186/s13063-022-07053-7.

## Introduction

Randomized trials rely on chance to form patient groups that are comparable at baseline. However, randomization balances the trial arms only in expectation, as a long term average; it does not guarantee that the groups will be comparable in any given instance [1–3]. As a result, current guidelines recommend that analyses of randomized clinical trials be adjusted for baseline patient characteristics that are associated with the outcome [4–6]. This approach assumes that the researchers are interested in the conditional treatment effect, i.e., treatment effect with all other patient characteristics held constant [7]. Several adjustment methods exist, including multiple regression, use of propensity scores, and other methods [8]. Here, we will consider only one case, adjustment for the confounder using logistic regression. In this case, as an added benefit, adjustment for prognostic factors will eliminate a conservative bias due to the non-collapsibility of the odds ratio, which occurs even when the trial arms are balanced [9–12].

Ideally, the adjustment model should represent correctly the effects of the prognostic factors under consideration. For example, if being 10 years older doubled the risk of death, this is the effect of age that should be used for adjustment. In real life, true effects are typically unknown, and the analyst estimates the effect of age from the trial sample at hand. But this sample-based model reflects the associations present in the study sample and will not necessarily yield the correct effect estimate—possibly, the effect of 10 years of age will be to triple the risk in this particular dataset, or to increase it by half, or even to reduce the risk. There is no guarantee that statistical adjustment based on available data will yield the correct estimate of the treatment effect, but it is also possible that the effect of over-fitting would be negligible.

To what extent using a potentially over-fitted sample-based adjustment model affects the estimation of treatment effects in randomized trials has not been explored to our knowledge. In this study, we use simulated data to compare a sample-based adjustment model to a true adjustment model, in terms of bias in estimating the treatment effect, as well as its variance, accuracy, and power.

## Methods

We conducted an experimental simulation study. In brief, in each iteration, we generated a clinical trial dataset in which a patient was either treated or untreated (1:1), and each was assigned a specific value of the potential confounder. A binary outcome variable was generated for each patient, and the trial results were analyzed using three logistic regression models: without adjustment for the potential confounder, with confounder adjustment using a sample-based model, and with confounder adjustment using the true confounder effect. The estimates of the treatment effect were compared in terms of bias, variance, proportion of treatment effects that were reasonably close to the true value, power, and type 1 error (when the modeled treatment effect was nil). Each experiment was replicated 50,000 times.

### Data generation

For each sample, we generated individual observations as follows: the treatment variable *T* was set to 1 in the experimental group and to 0 in the control group, and the potential confounder variable *C* was drawn either from a uniform distribution or from a Bernoulli distribution. We note that since *C* is independent of treatment under random allocation, it cannot be a confounder of the treatment effect in expectation (in other words, the *estimator* of the effect of treatment is unconfounded). However, *C* can cause “realized confounding” when by chance its distribution is not balanced across the two trial arms (i.e., any particular *estimate* of the effect of treatment can be confounded). Hereafter, for simplicity, we use the term “confounder” to designate a covariate *C* that is associated with the outcome and may be unbalanced between trial arms in any particular sample.

To facilitate comparisons between models, we selected the distributions of C so as to obtain the same variance. Thus, the uniform distribution of *C* had bounds − 0.75 and + 0.75 (variance was 1.5^2^/12, or 0.1875). The binary case had a Bernoulli parameter of 0.25 (variance was 0.25*0.75, or 0.1875). The expectations of *C* were 0 for the continuous case and 0.25 for the binary case.

Then, the probability of outcome r in an individual was obtained using the equation Logit(r) = *β*_1_*T* + *β*_2_*C*. The value of *β*_1_ was set to 0, 0.5, or 1.0 (we used positive values of *β*_1_ to facilitate the interpretation of the results; therefore, the outcome was clinically desirable). The value of *β*_2_ was set to 0, − 0.5, or − 1.0. We note that the sign of *β*_2_ is arbitrary and does not alter the estimation of the treatment effect. The value of *r* was obtained as e^*β*1*T* + *β*2*C*^/(1 + e^*β*1*T* + *β*2*C*^). The individual outcome was generated as a Bernoulli random variable *Y* with parameter *r*.

Sample sizes in each treatment arm were 50, 100, 200, 500, and 1000.

### Analysis of each replicate

We estimated the treatment effects using these three models:

- Unadjusted analysis: Logit(*Y*) = *b*_0_ + *b*_1_*T*
- Adjusted for *C* using the sample-based model: Logit(*Y*) = *b*_0_' + *b*_1_'*T* + *b*_2_'*C*
- Adjusted for *C* using the true effect: Logit(*Y*) = *b*_0_″ + *b*_1_″*T* + *β*_2_*C*

The unadjusted model was included as a point of reference, even though it was not required to answer the research question. The difference between the two adjusted models is that *b*_2_' was estimated from the data, whereas *β*_2_ took the value used in the simulation; the product *β*_2_*C was introduced as an offset variable into the regression model.

### Analysis of the simulated results

For each of the 90 experimental situations (3 treatment effects, 3 confounder effects, 2 types of confounder, 5 sample sizes) and the 3 models, we report the following results:

- Bias in the estimated treatment effect, i.e., the mean of *b*_1_—*β*_1_.
- Variance of the estimated treatment effect *b*_1_.
- Proportion of estimated treatment effects *b*_1_ within ± 0.1 of the true parameter value (on the odds ratio scale, this corresponds to intervals of 0.89 to 1.11 when when *β*_1_ = 0, 1.49 to 1.82 when *β*_1_ = 0.5, and 2.46 to 3.00 when *β*_1_ = 1).
- Proportion of treatment effects that were statistically significant (*p* < 0.05), i.e., type 1 error rate when *β*_1_ = 0, and power when *β*_1_ = 0.5 or 1.

Because some result patterns were similar across values of the treatment effect or confounder effect, we show herein only selected results.

To better understand the relationships between estimates of treatment effect, estimates of confounder effect, and confounder imbalance, we conducted the following analyses, for strong confounder and treatment effects (*β*_2_ =  − 1 and *β*_1_ = 1), at *N* = 2 × 50:

- Scatterplots of estimates of treatment effect in the three models (unadjusted, adjusted for *C* using the sample, adjusted for *C* using the true effect); for a continuous confounder.
- Scatterplots of observed estimates of the adjusted treatment effect *b*_1_ versus observed confounder effect *b*_2_, for both types of confounder, with non-parametric regression lines (Lowess).
- Scatterplots of observed estimates of the adjusted treatment effect *b*_1_ versus baseline imbalance between treatment arms in the confounder (using Cohen’s *d*), with non-parametric regression lines (Lowess).

The simulations and analyses were performed using the R software version R-4.0.2 (R Foundation for Statistical Computing, Vienna, Austria. URL https://www.R-project.org/).

## Results

All models converged in all 90 experimental conditions.

### Bias

When *C* was not associated with the outcome (*β*_2_ = 0), treatment effects were biased upward in small samples, somewhat more under sample-based adjustment than without adjustment or adjustment using the true model, for both a continuous and a binary confounder (Table 1). This upward bias was also detected in presence of confounding. Furthermore, in presence of potential confounding (*β*_2_ < 0), unadjusted estimates of treatment effect were biased downward, for both types of confounder, which corresponds to the expected effect of non-collapsibility of the odds ratio. Overall, adjustment using the true model (*β*_2_) produced less positive bias at small sample size than adjustment with the sample-based model (*b*_2_).

**Table 1:** Bias in the estimation of treatment effect (*β*_1_ = 1), for different values of the confounder effect (*β*_2_) and of sample size, for 3 logistic regression models: unadjusted for confounder, adjusted in sample-based model, and adjusted in true model

Sample size	Unadjusted analysis	Adjusted, sample-based model	Adjusted, true model	Unadjusted analysis	Adjusted, sample-based model	Adjusted, true model
	Continuous confounder effect *β*_2_ = 0			Binary confounder effect *β*_2_ = 0		
2 × 50	0.021	0.033	0.021	0.023	0.034	0.023
2 × 100	0.011	0.017	0.011	0.012	0.017	0.012
2 × 200	0.006	0.009	0.006	0.005	0.008	0.005
2 × 500	0.003	0.004	0.003	0.001	0.003	0.001
2 × 1000	0.000	0.001	0.000	0.000	0.001	0.000
	Continuous confounder effect *β*_2_ = − 0.5			Binary confounder effect *β*_2_ = − 0.5		
2 × 50	0.014	0.038	0.025	0.015	0.040	0.026
2 × 100	0.001	0.017	0.011	0.002	0.020	0.013
2 × 200	− 0.005	0.009	0.006	− 0.006	0.008	0.005
2 × 500	− 0.009	0.003	0.002	− 0.010	0.003	0.002
2 × 1000	− 0.009	0.002	0.001	− 0.010	0.002	0.001
	Continuous confounder effect *β*_2_ = − 1			Binary confounder effect *β*_2_ = − 1		
2 × 50	− 0.018	0.038	0.022	− 0.021	0.038	0.023
2 × 100	− 0.031	0.017	0.010	− 0.034	0.019	0.011
2 × 200	− 0.037	0.008	0.005	− 0.040	0.008	0.004
2 × 500	− 0.039	0.004	0.003	− 0.043	0.003	0.002
2 × 1000	− 0.040	0.002	0.001	− 0.044	0.002	0.001

### Variance

We limit variance results to simulations under a strong confounder effect (*β*_2_ =  − 1), as the patterns were similar but weaker for the lower value of *β*_2_ (Table 2). The variance of the treatment effect decreased predictably with sample size and was slightly lower in the unadjusted analyses. The two adjustment methods performed similarly.

**Table 2:** Variance in the estimation of treatment effect (*b*_1_), for different values of the true treatment effect (*β*_1_) and of sample size, with a strong confounder effect (*β*_2_ =  − 1): unadjusted for confounder, adjusted in sample-based model, and adjusted in true model

Sample size	Unadjusted analysis	Adjusted, sample-based model	Adjusted, true model	Unadjusted analysis	Adjusted, sample-based model	Adjusted, true model
	Treatment effect *β*_1_ = 0, continuous confounder			Treatment effect *β*_1_ = 0, binary confounder		
2 × 50	0.17	0.18	0.18	0.17	0.18	0.18
2 × 100	0.08	0.09	0.09	0.08	0.09	0.09
2 × 200	0.04	0.04	0.04	0.04	0.04	0.04
2 × 500	0.02	0.02	0.02	0.02	0.02	0.02
2 × 1000	0.01	0.01	0.01	0.01	0.01	0.01
	Treatment effect *β*_1_ = 0.5, continuous confounder			Treatment effect *β*_1_ = 0.5, binary confounder		
2 × 50	0.17	0.19	0.18	0.17	0.18	0.18
2 × 100	0.08	0.09	0.09	0.08	0.09	0.09
2 × 200	0.04	0.04	0.04	0.04	0.04	0.04
2 × 500	0.02	0.02	0.02	0.02	0.02	0.02
2 × 1000	0.01	0.01	0.01	0.01	0.01	0.01
	Treatment effect *β*_1_ = 1, continuous confounder			Treatment effect *β*_1_ = 1, binary confounder		
2 × 50	0.19	0.21	0.20	0.18	0.20	0.19
2 × 100	0.09	0.10	0.10	0.09	0.10	0.09
2 × 200	0.05	0.05	0.05	0.04	0.05	0.05
2 × 500	0.02	0.02	0.02	0.02	0.02	0.02
2 × 1000	0.01	0.01	0.01	0.01	0.01	0.01

### Accuracy of estimation

Proportions of estimates that fell within ± 0.1 of the real parameter value were fairly low and even for the largest sample size of 2 × 1000 they barely reached 70% (Table 3). Unadjusted analyses produced less accurate estimates when the treatment effect was strong, which is consistent with the conservative bias of the estimates due to non-collapsibility. The two adjustment methods performed similarly.

**Table 3:** Proportion of treatment effects within ± 0.1 of the true value, for different values of the true treatment effect (*β*_1_) and of sample size, with a strong confounder effect (*β*_2_ =  − 1): unadjusted for confounder, adjusted in sample-based model, and adjusted in true model

Sample size	Unadjusted analysis	Adjusted, sample-based model	Adjusted, true model	Unadjusted analysis	Adjusted, sample-based model	Adjusted, true model
	Treatment effect *β*_1_ = 0, continuous confounder			Treatment effect *β*_1_ = 0, binary confounder		
2 × 50	0.23	0.19	0.19	0.24	0.19	0.19
2 × 100	0.28	0.27	0.27	0.27	0.26	0.26
2 × 200	0.34	0.37	0.37	0.35	0.37	0.37
2 × 500	0.57	0.56	0.56	0.58	0.56	0.56
2 × 1000	0.73	0.73	0.73	0.73	0.72	0.72
	Treatment effect *β*_1_ = 0.5, continuous confounder			Treatment effect *β*_1_ = 0.5, binary confounder		
2 × 50	0.23	0.18	0.19	0.24	0.19	0.19
2 × 100	0.28	0.27	0.27	0.28	0.27	0.27
2 × 200	0.38	0.37	0.37	0.38	0.37	0.37
2 × 500	0.55	0.55	0.55	0.57	0.56	0.56
2 × 1000	0.71	0.72	0.72	0.72	0.72	0.72
	Treatment effect *β*_1_ = 1, continuous confounder			Treatment effect *β*_1_ = 1, binary confounder		
2 × 50	0.18	0.17	0.18	0.19	0.18	0.18
2 × 100	0.26	0.25	0.25	0.26	0.26	0.26
2 × 200	0.35	0.35	0.36	0.36	0.36	0.36
2 × 500	0.53	0.53	0.54	0.52	0.54	0.54
2 × 1000	0.66	0.70	0.70	0.66	0.70	0.71

### Type 1 error and power

Type 1 errors were well controlled in all circumstances (Table 4). Power rose predictably with sample size and was slightly better for adjusted analyses than for unadjusted analyses (Table 5). The two adjustment methods yielded similar power.

**Table 4:** Proportion of type 1 errors, for different values of the confounder effect (*β*_2_) and of sample size, for 3 logistic regression models: unadjusted for confounder, adjusted in sample-based model, and adjusted in true model

Sample size	Unadjusted analysis	Adjusted, sample-based model	Adjusted, true model	Unadjusted analysis	Adjusted, sample-based model	Adjusted, true model
	Continuous confounder effect *β*_2_ = 0			Binary confounder effect *β*_2_ = 0		
2 × 50	0.056	0.053	0.056	0.056	0.052	0.056
2 × 100	0.056	0.053	0.056	0.055	0.051	0.055
2 × 200	0.050	0.050	0.050	0.052	0.051	0.052
2 × 500	0.049	0.050	0.049	0.050	0.051	0.050
2 × 1000	0.050	0.049	0.050	0.050	0.049	0.050
	Continuous confounder effect *β*_2_ = − 0.5			Binary confounder effect *β*_2_ = − 0.5		
2 × 50	0.057	0.052	0.050	0.056	0.052	0.050
2 × 100	0.055	0.050	0.049	0.056	0.052	0.050
2 × 200	0.051	0.050	0.050	0.050	0.049	0.048
2 × 500	0.048	0.048	0.048	0.052	0.050	0.050
2 × 1000	0.053	0.051	0.051	0.051	0.051	0.050
	Continuous confounder effect *β*_2_ = − 1			Binary confounder effect *β*_2_ = − 1		
2 × 50	0.059	0.051	0.050	0.056	0.051	0.050
2 × 100	0.057	0.051	0.051	0.053	0.049	0.048
2 × 200	0.052	0.051	0.051	0.049	0.050	0.050
2 × 500	0.049	0.051	0.050	0.050	0.048	0.048
2 × 1000	0.053	0.051	0.051	0.050	0.049	0.049

**Table 5:** Observed power for different values of the treatment effect (*β*_1_) and of sample size, with a strong confounder effect (*β*_2_ =  − 1), for 3 logistic regression models: unadjusted for confounder, adjusted in sample-based model, and adjusted in true model

Sample size	Unadjusted analysis	Adjusted, sample-based model	Adjusted, true model	Unadjusted analysis	Adjusted, sample-based model	Adjusted, true model
	Treatment effect *β*_1_ = 0.5, continuous confounder			Treatment effect *β*_1_ = 0.5, binary confounder		
2 × 50	0.24	0.23	0.23	0.24	0.22	0.22
2 × 100	0.40	0.40	0.40	0.41	0.40	0.40
2 × 200	0.66	0.68	0.68	0.67	0.68	0.68
2 × 500	0.96	0.97	0.97	0.96	0.97	0.97
2 × 1000	1.00	1.00	1.00	1.00	1.00	1.00
	Treatment effect *β*_1_ = 1, continuous confounder			Treatment effect *β*_1_ = 1, binary confounder		
2 × 50	0.64	0.65	0.65	0.67	0.67	0.67
2 × 100	0.90	0.91	0.91	0.92	0.92	0.92
2 × 200	1.00	1.00	1.00	1.00	1.00	1.00
2 × 500	1.00	1.00	1.00	1.00	1.00	1.00
2 × 1000	1.00	1.00	1.00	1.00	1.00	1.00

### Correlations between treatment effect estimates

**Fig. 1:**
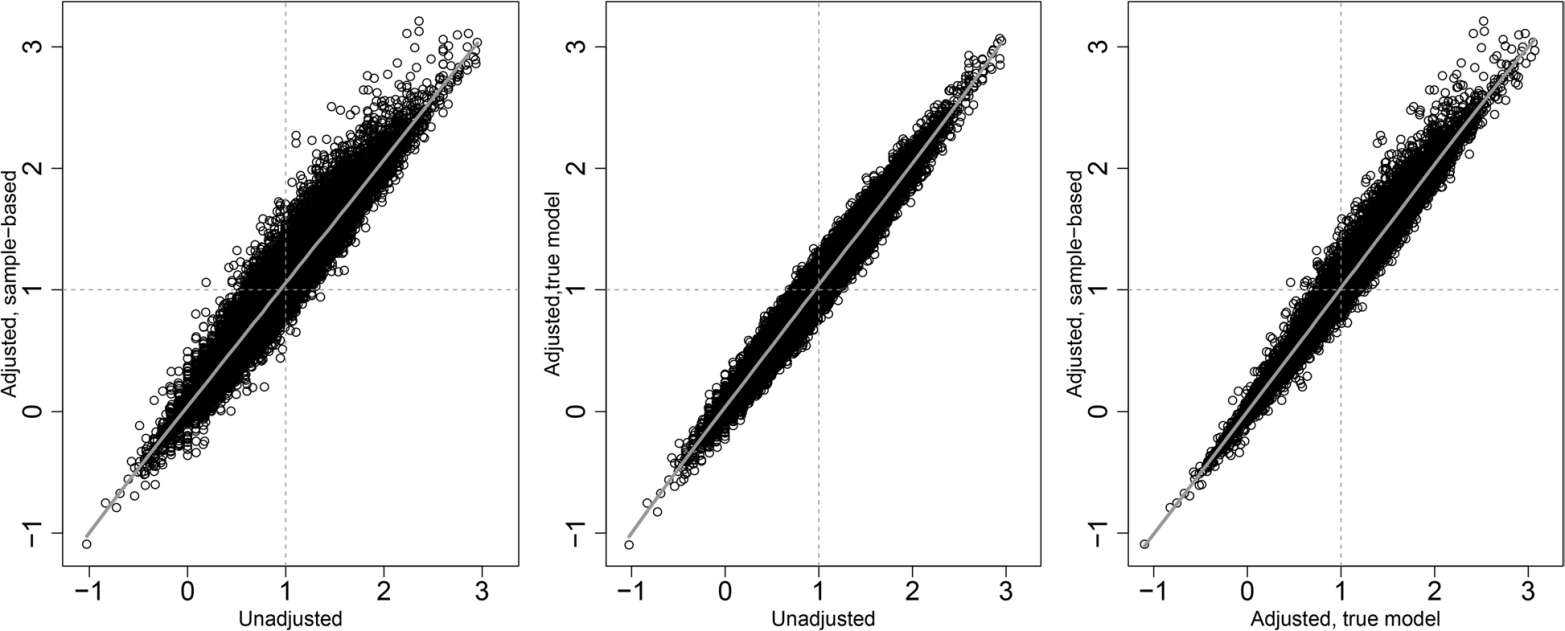
Scatterplots of unadjusted and adjusted estimates of treatment effect (*b*_1_), for a strong treatment effect (*β*_1_ = 1), a strong continuous confounder (*β*_2_ =  − 1), and sample size 2 × 50

Unadjusted estimates of treatment effect were more strongly correlated with estimates adjusted for the true effect than with sample-based adjustment (Fig. 1). The Pearson correlation coefficients were 0.97, 0.98, and 0.99 in the three panels of Fig. 1. Despite the high correlations, the differences between the estimates of treatment effect could vary by 0.5 or 1 unit (on the logit scale) in some samples.

### Joint distributions of estimated adjusted treatment and confounder effects

**Fig. 2:**
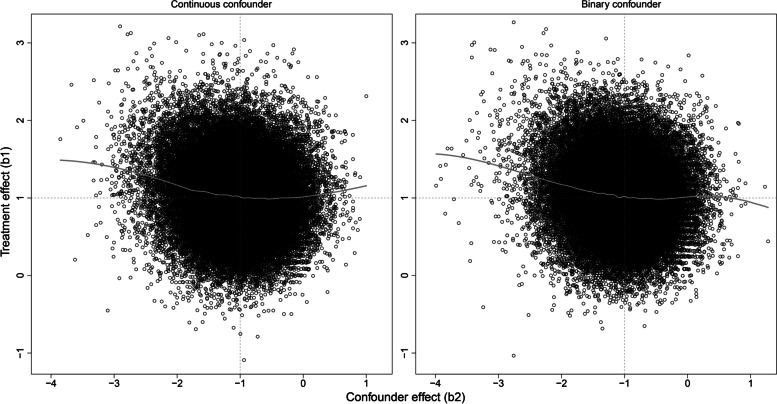
Scatter plots of (sample-based) adjusted estimates of the treatment effect (*b*_1_) as a function of the estimated confounder effect (*b*_2_), with true parameter values *β*_1_ = 1 and *β*_2_ =  − 1, for continuous and binary confounders, at sample size 2 × 50. Grey lines represent non-parametric regression functions (Lowess)

The scatterplots of the estimated treatment and confounder effects at size 2 × 50 (Fig. 2) yielded similar results for continuous and binary confounders. The estimated confounder effect *b*_2_ ranged between approximately − 3 and 1, for a true parameter value of − 1. Treatment effects *b*_1_ appeared stronger at negative values of the estimated confounder effect (i.e., when the confounder effect was overestimated). This showed as an asymmetry of the scatterplots and was confirmed by the non-parametric regression lines. Pearson correlation coefficients between the confounder effect and the treatment effect were − 0.10 for both types of confounder.

### Estimated treatment effects as a function of baseline imbalance

**Fig. 3:**
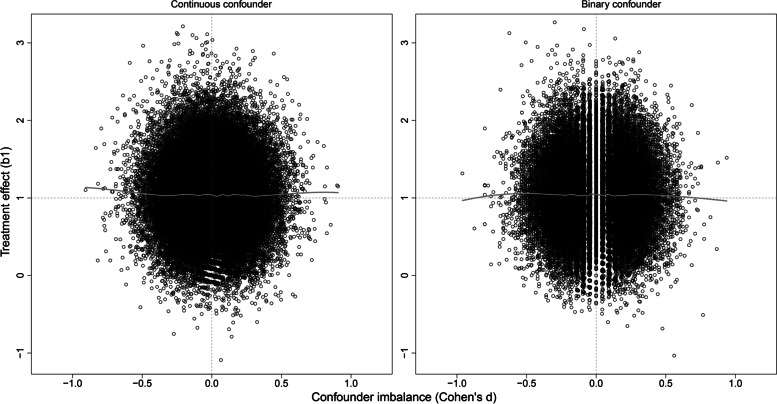
Scatter plot of (sample based) adjusted estimates of the treatment effect (*b*_1_) as a function of the confounder imbalance between the two groups (Cohen’s *d*), with true parameter values *β*_1_ = 1 and *β*_2_ =  − 1, for continuous and binary confounders, at sample size 2 × 50. Grey lines represent non-parametric regression functions (Lowess)

The scatterplots of the estimated adjusted treatment effect as a function of baseline confounder imbalance were symmetric and did not reveal any bias or obvious heteroscedasticity (Fig. 3). Results were similar for continuous and binary confounders. Cohen’s *d*—i.e., between-arm difference in *C* expressed in pooled observed standard deviation units—ranged from approximately − 0.6 to + 0.6 (since confounder variance was 0.1875 by design, one standard deviation unit was 0.4330, and 0.6 of this value corresponds to 0.26). Pearson correlation coefficients between the confounder effect and the treatment effect were null in both scenarios.

## Discussion

This simulation study indicated that a sample-based adjustment model has only a small disadvantage vis-à-vis a true model when analyzing the results of a clinical trial. Specifically, the sample-based model produced estimates of the treatment effect that were more positively biased, but only at small sample sizes (2 × 50). There were no losses in terms of accuracy, type 1 error, or power. Furthermore, we found no relation between the magnitude in the baseline imbalance in the potential confounder and the estimation of the treatment effect, after sample-based adjustment. This indicates that sample-based adjustment works adequately across levels of imbalance. We found that the adjusted treatment effect was overestimated when the effect of the confounder was over-estimated as well, but this occurred only in rather extreme situations (observed confounder effect at least twice as strong as the true effect). Overall, these results are reassuring; the current practice of adjusting based on the sample at hand appears reasonable.

We used the true adjustment model as a yardstick to demonstrate the possible impact of an incorrectly estimated sample-based confounder effect, but in real-life, the true confounder effect *β*_2_ is usually unknown. Reasonably solid estimates may exist for some confounder effects: e.g., the prediction of death following brain injury has been modeled in trials that enrolled thousands of patients [13], and various mortality prediction models are available for intensive care patients, patients hospitalized with COVID-19, patients with coronary artery disease, etc. In other instances reasonable guesses are possible, at least as to the direction of the effect—e.g., greater severity of disease, presence of comorbidities, or older age are typically associated with less favorable outcomes. If the observed associations ran in the opposite direction, it may be prudent either to remove the paradoxical covariate from the adjustment model (effectively setting the regression coefficient to 0) or to apply other regularization methods. In any case, such adaptive procedures should be pre-specified in the statistical analysis plan, to avoid post hoc selection of the main analysis model.

This simulation study also confirmed two established results. One is the conservative bias present in unadjusted analyses of binary outcomes, due to the non-collapsibility of the odds ratio [9–12]. This bias increases with the effect of the confounding factor under consideration. This confirms the utility of adjusting trial results for known risk factors regardless of any imbalance at baseline. Such adjustment was particularly useful at larger sample sizes; indeed, with 2 × 1000 observations, adjusted estimates were substantially more accurate than unadjusted estimates.

The other confirmatory result was the positive bias of logistic regression coefficient estimates at small sample sizes. This too has been described previously [14, 15]. This bias away from the null in small samples was revealed by the adjustment procedures, and this is one area where the true adjustment model performed better than sample-based adjustment.

Finally, we did not observe any gain of power in adjusted models, compared with unadjusted analyses. This too is consistent with current knowledge. Power gains from confounder adjustment are expected in linear regression models for continuous outcomes, but not necessarily in analyses of binary outcomes [16], as adjusted estimates of treatment effect are generally less biased toward the null but also less precise. This was also shown in a previous simulation study partly based on actual trial results [17].

While estimates of treatment effect and of sample-based confounder effect were only weakly correlated, a notable bias in the treatment effect was seen only when the confounder effect was overestimated (Fig. 2). This suggest that analysts should remain cautious when the confounder effect is much larger than expected, based on prior knowledge. Furthermore, while unadjusted and adjusted treatment effects were highly correlated, substantial differences occurred on occasion (Fig. 1). This indicated that data dredging has the potential for yielding spurious results and reinforces the recommendation that adjustment models be always pre-specified.

A limitation of this study is that we did not explore all possible situations, such as different levels of baseline risk, or multiple adjustment variables. However, we believe that this simulation study provides a realistic assessment of the potential of true adjustment models to improve the analysis strategy for clinical trials. We found this potential to be minor; the risk inherent in relying on sample-based models seems negligible.

Another limitation is that we did not examine what happens if the treatment effect varies across subgroups (i.e., effect-modification, assuming that the effect-modifier is distinct from the confounder). If the effect-modifier is measured, then stratum-specific estimates of *β*_1_ can be obtained, with adjustment for the potential confounder. However, the estimation of the confounder effect can be pooled over strata of the effect-modifier, which may reduce potential overfitting, according to our results. This would particularly benefit the estimation of treatment effects in small strata.

In conclusion, we saw on average little or no disadvantage to using a sample-based model, rather than a true regression model, for the adjustment for baseline prognostic factors. Adjusted estimates performed better than unadjusted estimates.

## Supplementary Information

**Additional file 1: Appendix.** R code used for simulations.

## Data Availability

Not applicable (the simulated datasets were discarded).
