# Supplementary material for: Adjustment for baseline characteristics in randomized trials using logistic regression: sample-based model versus true model
Source: Trials. 2023 Feb 13;24:107. doi: 10.1186/s13063-022-07053-7 (PMC9924183; doi:10.1186/s13063-022-07053-7)
Supplement: Supplementary file 1 — Additional file 1: Appendix. R code used for simulations. [file 13063_2022_7053_MOESM1_ESM.docx]

APPENDIX: R code used for simulations

library(pROC)

set.seed(24)

# INPUT of parameters

NBSIMU<- 50000

Beta1 <- c(0, 0.5, 1)

Beta2 <- c(0, -0.5, -1)

PREV <- 0.25 # prevalence of binary C = 25% !!! (e.g. Tobacco)

Ngr <- c(50,100,200,500,1000)

# create array results

# dimension 1: simulation number

# dimension 2: output type

# converged : model converged (TRUE / FALSE)

# Outcome_Homogene: identical outcome in whole arm

# pvalue : p for beta 1

# bias : difference between estimate and estimand beta 1

# beta0 : intercept

# beta1 : estimated b1

# beta2 : estimated b2

# cohen.d : baseline difference in C (standardaized)

# dimension 3: value of Beta 1

# dimension 4: value of Beta 2

# dimension 5: sample size (per group)

# dimension 6: adjustment strategy (1=none, 2=sample based, 3=true model)

OUTPUT <- c("converged", "Outcome_Homogene", "pvalue", "bias", "beta0", "beta1", "beta2", "cohen.d", "auc", "auc_outcome", "prop_C", "prop_I", "risk_C", "risk_I", "logit_C", "logit_I")

STRATEGIE <- c("Strategie1", "Strategie2", "Strategie3")

resCovar.C <- array(NA,

dim=c(NBSIMU, length(OUTPUT), length(Beta1), length(Beta2), length(Ngr), length(STRATEGIE)),

dimnames=list(

c(1:NBSIMU),

OUTPUT,

as.character(Beta1),

as.character(Beta2),

as.character(Ngr),

as.character(STRATEGIE)))

resCovar.B <- array(NA,

dim=c(NBSIMU, length(OUTPUT), length(Beta1), length(Beta2), length(Ngr), length(STRATEGIE)),

dimnames=list(

c(1:NBSIMU),

OUTPUT,

as.character(Beta1),

as.character(Beta2),

as.character(Ngr),

as.character(STRATEGIE)))

print(Sys.time())

for (nn in 1:length(Ngr)) {

for(bb2 in 1:length(Beta2)) {

print(paste("sample size: ", Ngr[nn], "; Beta2 = ", Beta2[bb2], sep=""))

for (bb in 1:length(Beta1)){

for(i in 1:NBSIMU){

# On simule l'echantillon

DATA <- NULL

DATA$Trt <- rep(c(0,1), each=Ngr[nn])

DATA$Covar.C <- runif(2*Ngr[nn], -0.75, 0.75) # extrema to achieve variance of 0.1875 (+/- 0.75)

DATA$logit.C <- Beta1[bb]*DATA$Trt + Beta2[bb2]*DATA$Covar.C

DATA$Proba.C <- exp(DATA$logit.C)/(1+exp(DATA$logit.C))

DATA$OUTCOME.C <- rbinom(n=2*Ngr[nn], size=1, prob=DATA$Proba.C)

DATA$Covar.B <- rbinom(n=2*Ngr[nn], size=1, prob=PREV)

DATA$logit.B <- Beta1[bb]*DATA$Trt + Beta2[bb2]*DATA$Covar.B

DATA$Proba.B <- exp(DATA$logit.B)/(1+exp(DATA$logit.B))

DATA$OUTCOME.B <- rbinom(n=2*Ngr[nn], size=1, prob=DATA$Proba.B)

DATA <- as.data.frame(DATA[c("Trt", "OUTCOME.C", "Covar.C", "Proba.C", "logit.C", "OUTCOME.B", "Covar.B", "Proba.B", "logit.B")])

X_MAT1 <- as.data.frame(cbind(rep(1,2*Ngr[nn]), DATA[c("Trt", "Covar.C")]))

names(X_MAT1)[1] <- "Intercept"

X_MAT0 <- X_MAT1[,-3]

X_MAT2 <- X_MAT1

X_MAT2[,3] <- Beta2[bb2] * I(DATA$Covar.C)

# modelisation 1: no adjustment

MODEL1 <- glm.fit(X_MAT0, DATA$OUTCOME.C, family=binomial(link='logit'))

# modelisation 2: sample-based adjustment

MODEL2 <- glm.fit(X_MAT1, DATA$OUTCOME.C, family=binomial(link='logit'))

# modelisation 3: true model adjustment

MODEL3 <- glm.fit(X_MAT0, DATA$OUTCOME.C, offset=X_MAT2[,3], family=binomial(link='logit'))

resCovar.C[i,"converged",bb,bb2,nn,1] <- MODEL1$converged

resCovar.C[i,"converged",bb,bb2,nn,2] <- MODEL2$converged

resCovar.C[i,"converged",bb,bb2,nn,3] <- MODEL3$converged

resCovar.C[i,"Outcome_Homogene",bb,bb2,nn,] <- var(DATA$OUTCOME.C[1:Ngr[nn]])==0 | var(DATA$OUTCOME.C[Ngr[nn]+1:Ngr[nn]])==0

resCovar.C[i,"pvalue",bb,bb2,nn,1] <- summary.glm(MODEL1)$coef[2,4]

resCovar.C[i,"pvalue",bb,bb2,nn,2] <- summary.glm(MODEL2)$coef[2,4]

resCovar.C[i,"pvalue",bb,bb2,nn,3] <- summary.glm(MODEL3)$coef[2,4]

resCovar.C[i,"bias",bb,bb2,nn,1] <- coefficients(MODEL1)[2] - Beta1[bb]

resCovar.C[i,"bias",bb,bb2,nn,2] <- coefficients(MODEL2)[2] - Beta1[bb]

resCovar.C[i,"bias",bb,bb2,nn,3] <- coefficients(MODEL3)[2] - Beta1[bb]

resCovar.C[i,"beta0",bb,bb2,nn,1] <- coefficients(MODEL1)[1]

resCovar.C[i,"beta0",bb,bb2,nn,2] <- coefficients(MODEL2)[1]

resCovar.C[i,"beta0",bb,bb2,nn,3] <- coefficients(MODEL3)[1]

resCovar.C[i,"beta1",bb,bb2,nn,1] <- coefficients(MODEL1)[2]

resCovar.C[i,"beta1",bb,bb2,nn,2] <- coefficients(MODEL2)[2]

resCovar.C[i,"beta1",bb,bb2,nn,3] <- coefficients(MODEL3)[2]

resCovar.C[i,"beta2",bb,bb2,nn,2] <- coefficients(MODEL2)[3]

PooledSD <- sqrt(( (Ngr[nn] - 1) * var(DATA$Covar.C[1:Ngr[nn]]) + (Ngr[nn] - 1) * var(DATA$Covar.C[Ngr[nn]+1:Ngr[nn]]) ) / (2*Ngr[nn] - 2) )

resCovar.C[i,"cohen.d",bb,bb2,nn,] <- ( mean(DATA$Covar.C[1:Ngr[nn]]) - mean(DATA$Covar.C[Ngr[nn]+1:Ngr[nn]]) ) / PooledSD

# idem avec Covar.B

X_MAT1.B <- as.data.frame(cbind(rep(1,2*Ngr[nn]), DATA[c("Trt", "Covar.B")]))

names(X_MAT1.B)[1] <- "Intercept"

X_MAT0.B <- X_MAT1.B[,-3]

X_MAT2.B <- X_MAT1.B

X_MAT2.B[,3] <- Beta2[bb2] * I(DATA$Covar.B)

# modelisation 1: no adjustment

MODEL1.B <- glm.fit(X_MAT0.B, DATA$OUTCOME.B, family=binomial(link='logit'))

# modelisation 2: sample-based adjustment

MODEL2.B <- glm.fit(X_MAT1.B, DATA$OUTCOME.B, family=binomial(link='logit'))

# modelisation 3: true model adjustment

MODEL3.B <- glm.fit(X_MAT0.B, DATA$OUTCOME.B, offset=X_MAT2.B[,3], family=binomial(link='logit'))

resCovar.B[i,"converged",bb,bb2,nn,1] <- MODEL1.B$converged

resCovar.B[i,"converged",bb,bb2,nn,2] <- MODEL2.B$converged

resCovar.B[i,"converged",bb,bb2,nn,3] <- MODEL3.B$converged

resCovar.B[i,"Outcome_Homogene",bb,bb2,nn,] <- var(DATA$OUTCOME.B[1:Ngr[nn]])==0 | var(DATA$OUTCOME.B[Ngr[nn]+1:Ngr[nn]])==0

resCovar.B[i,"pvalue",bb,bb2,nn,1] <- summary.glm(MODEL1.B)$coef[2,4]

resCovar.B[i,"pvalue",bb,bb2,nn,2] <- summary.glm(MODEL2.B)$coef[2,4]

resCovar.B[i,"pvalue",bb,bb2,nn,3] <- summary.glm(MODEL3.B)$coef[2,4]

resCovar.B[i,"bias",bb,bb2,nn,1] <- coefficients(MODEL1.B)[2] - Beta1[bb]

resCovar.B[i,"bias",bb,bb2,nn,2] <- coefficients(MODEL2.B)[2] - Beta1[bb]

resCovar.B[i,"bias",bb,bb2,nn,3] <- coefficients(MODEL3.B)[2] - Beta1[bb]

resCovar.B[i,"beta0",bb,bb2,nn,1] <- coefficients(MODEL1.B)[1]

resCovar.B[i,"beta0",bb,bb2,nn,2] <- coefficients(MODEL2.B)[1]

resCovar.B[i,"beta0",bb,bb2,nn,3] <- coefficients(MODEL3.B)[1]

resCovar.B[i,"beta1",bb,bb2,nn,1] <- coefficients(MODEL1.B)[2]

resCovar.B[i,"beta1",bb,bb2,nn,2] <- coefficients(MODEL2.B)[2]

resCovar.B[i,"beta1",bb,bb2,nn,3] <- coefficients(MODEL3.B)[2]

resCovar.B[i,"beta2",bb,bb2,nn,2] <- coefficients(MODEL2.B)[3]

PooledSD.B <- sqrt(( (Ngr[nn] - 1) * var(DATA$Covar.B[1:Ngr[nn]]) + (Ngr[nn] - 1) * var(DATA$Covar.B[Ngr[nn]+1:Ngr[nn]]) ) / (2*Ngr[nn] - 2) )

resCovar.B[i,"cohen.d",bb,bb2,nn,] <- ( mean(DATA$Covar.B[1:Ngr[nn]]) - mean(DATA$Covar.B[Ngr[nn]+1:Ngr[nn]]) ) / PooledSD.B

}

}

}

}

print(Sys.time())

######################################################################################################

# Objects containing vrious results !!

RESULT <- c("no_converg", "pb_homogen", "pb_ajust", "mean_bias", "var_bias",

"MSE", "prop0.1_OK", "prop0.2_OK", "power")

resultat.C <- array(NA,

dim=c(length(RESULT), length(Beta1), length(Beta2), length(Ngr), length(STRATEGIE)),

dimnames=list(

RESULT,

as.character(Beta1),

as.character(Beta2),

as.character(Ngr),

as.character(STRATEGIE)))

resultat.B <- array(NA,

dim=c(length(RESULT), length(Beta1), length(Beta2), length(Ngr), length(STRATEGIE)),

dimnames=list(

RESULT,

as.character(Beta1),

as.character(Beta2),

as.character(Ngr),

as.character(STRATEGIE)))

# functions declaration

myprop <- function(x, betaa1) { length(x[x<0.8*betaa1 & x>1.2*betaa1]) / length(x) }

mypropbis <- function(x, betaa1) { length(x[x<(betaa1+0.1) & x>(betaa1-0.1)]) / length(x) }

mypropbis2<- function(x, betaa1) { length(x[x<(betaa1+0.2) & x>(betaa1-0.2)]) / length(x) }

pvalLess.05<-function(x) {mean(x<0.05,na.rm=T)}

for (nn in 1:length(Ngr)){

for(bb in 1:length(Beta1)){

for(bb2 in 1:length(Beta2)){

resultat.C["no_converg",bb,bb2,nn,1] <- sum(resCovar.C[,"converged",bb,bb2,nn,1]==FALSE)

resultat.C["no_converg",bb,bb2,nn,2] <- sum(resCovar.C[,"converged",bb,bb2,nn,2]==FALSE)

resultat.C["no_converg",bb,bb2,nn,3] <- sum(resCovar.C[,"converged",bb,bb2,nn,3]==FALSE)

resultat.C["pb_homogen",bb,bb2,nn,1] <- sum(resCovar.C[,"Outcome_Homogene",bb,bb2,nn,1]==TRUE)

resultat.C["pb_homogen",bb,bb2,nn,2] <- sum(resCovar.C[,"Outcome_Homogene",bb,bb2,nn,2]==TRUE)

resultat.C["pb_homogen",bb,bb2,nn,3] <- sum(resCovar.C[,"Outcome_Homogene",bb,bb2,nn,3]==TRUE)

resultat.C["pb_ajust",bb,bb2,nn,] <- sum(resCovar.C[,"beta1",bb,bb2,nn,2]>18) - sum(resCovar.C[,"Outcome_Homogene",bb,bb2,nn,2]==TRUE) - sum(resCovar.C[,"converged",bb,bb2,nn,2]==FALSE) # problème (beta1 >18) post-hoc lié à l'ajustement en dépit d'une non-homogénéité de l'outcome selon TTT

resultat.C["mean_bias",bb,bb2,nn,1] <- mean(resCovar.C[,"bias",bb,bb2,nn,1][resCovar.C[,"converged",bb,bb2,nn,1]==TRUE])

resultat.C["mean_bias",bb,bb2,nn,2] <- mean(resCovar.C[,"bias",bb,bb2,nn,2][resCovar.C[,"converged",bb,bb2,nn,2]==TRUE])

resultat.C["mean_bias",bb,bb2,nn,3] <- mean(resCovar.C[,"bias",bb,bb2,nn,3][resCovar.C[,"converged",bb,bb2,nn,3]==TRUE])

resultat.C["var_bias",bb,bb2,nn,1] <- var(resCovar.C[,"bias",bb,bb2,nn,1][resCovar.C[,"converged",bb,bb2,nn,1]==TRUE])

resultat.C["var_bias",bb,bb2,nn,2] <- var(resCovar.C[,"bias",bb,bb2,nn,2][resCovar.C[,"converged",bb,bb2,nn,2]==TRUE])

resultat.C["var_bias",bb,bb2,nn,3] <- var(resCovar.C[,"bias",bb,bb2,nn,3][resCovar.C[,"converged",bb,bb2,nn,3]==TRUE])

resultat.C["MSE",bb,bb2,nn,1] <- mean(resCovar.C[,"bias",bb,bb2,nn,1][resCovar.C[,"converged",bb,bb2,nn,1]==TRUE]**2)

resultat.C["MSE",bb,bb2,nn,2] <- mean(resCovar.C[,"bias",bb,bb2,nn,2][resCovar.C[,"converged",bb,bb2,nn,2]==TRUE]**2)

resultat.C["MSE",bb,bb2,nn,3] <- mean(resCovar.C[,"bias",bb,bb2,nn,3][resCovar.C[,"converged",bb,bb2,nn,3]==TRUE]**2)

resultat.C["prop0.1_OK",bb,bb2,nn,1] <- mypropbis(resCovar.C[,"beta1",bb,bb2,nn,1][resCovar.C[,"converged",bb,bb2,nn,1]==TRUE], Beta1[bb])

resultat.C["prop0.1_OK",bb,bb2,nn,2] <- mypropbis(resCovar.C[,"beta1",bb,bb2,nn,2][resCovar.C[,"converged",bb,bb2,nn,2]==TRUE], Beta1[bb])

resultat.C["prop0.1_OK",bb,bb2,nn,3] <- mypropbis(resCovar.C[,"beta1",bb,bb2,nn,3][resCovar.C[,"converged",bb,bb2,nn,3]==TRUE], Beta1[bb])

resultat.C["prop0.2_OK",bb,bb2,nn,1] <- mypropbis2(resCovar.C[,"beta1",bb,bb2,nn,1][resCovar.C[,"converged",bb,bb2,nn,1]==TRUE], Beta1[bb])

resultat.C["prop0.2_OK",bb,bb2,nn,2] <- mypropbis2(resCovar.C[,"beta1",bb,bb2,nn,2][resCovar.C[,"converged",bb,bb2,nn,2]==TRUE], Beta1[bb])

resultat.C["prop0.2_OK",bb,bb2,nn,3] <- mypropbis2(resCovar.C[,"beta1",bb,bb2,nn,3][resCovar.C[,"converged",bb,bb2,nn,3]==TRUE], Beta1[bb])

resultat.C["power",bb,bb2,nn,1] <- pvalLess.05(resCovar.C[,"pvalue",bb,bb2,nn,1][resCovar.C[,"converged",bb,bb2,nn,1]==TRUE])

resultat.C["power",bb,bb2,nn,2] <- pvalLess.05(resCovar.C[,"pvalue",bb,bb2,nn,2][resCovar.C[,"converged",bb,bb2,nn,2]==TRUE])

resultat.C["power",bb,bb2,nn,3] <- pvalLess.05(resCovar.C[,"pvalue",bb,bb2,nn,3][resCovar.C[,"converged",bb,bb2,nn,3]==TRUE])

# idem for binary covariate

resultat.B["no_converg",bb,bb2,nn,1] <- sum(resCovar.B[,"converged",bb,bb2,nn,1]==FALSE)

resultat.B["no_converg",bb,bb2,nn,2] <- sum(resCovar.B[,"converged",bb,bb2,nn,2]==FALSE)

resultat.B["no_converg",bb,bb2,nn,3] <- sum(resCovar.B[,"converged",bb,bb2,nn,3]==FALSE)

resultat.B["pb_homogen",bb,bb2,nn,1] <- sum(resCovar.B[,"Outcome_Homogene",bb,bb2,nn,1]==TRUE)

resultat.B["pb_homogen",bb,bb2,nn,2] <- sum(resCovar.B[,"Outcome_Homogene",bb,bb2,nn,2]==TRUE)

resultat.B["pb_homogen",bb,bb2,nn,3] <- sum(resCovar.B[,"Outcome_Homogene",bb,bb2,nn,3]==TRUE)

resultat.B["pb_ajust",bb,bb2,nn,] <- sum(resCovar.B[,"beta1",bb,bb2,nn,2]>18) - sum(resCovar.B[,"Outcome_Homogene",bb,bb2,nn,2]==TRUE) - sum(resCovar.B[,"converged",bb,bb2,nn,2]==FALSE) # problème (beta1 >18) post-hoc lié à l'ajustement en dépit d'une non-homogénéité de l'outcome selon TTT

resultat.B["mean_bias",bb,bb2,nn,1] <- mean(resCovar.B[,"bias",bb,bb2,nn,1][resCovar.B[,"converged",bb,bb2,nn,1]==TRUE])

resultat.B["mean_bias",bb,bb2,nn,2] <- mean(resCovar.B[,"bias",bb,bb2,nn,2][resCovar.B[,"converged",bb,bb2,nn,2]==TRUE])

resultat.B["mean_bias",bb,bb2,nn,3] <- mean(resCovar.B[,"bias",bb,bb2,nn,3][resCovar.B[,"converged",bb,bb2,nn,3]==TRUE])

resultat.B["var_bias",bb,bb2,nn,1] <- var(resCovar.B[,"bias",bb,bb2,nn,1][resCovar.B[,"converged",bb,bb2,nn,1]==TRUE])

resultat.B["var_bias",bb,bb2,nn,2] <- var(resCovar.B[,"bias",bb,bb2,nn,2][resCovar.B[,"converged",bb,bb2,nn,2]==TRUE])

resultat.B["var_bias",bb,bb2,nn,3] <- var(resCovar.B[,"bias",bb,bb2,nn,3][resCovar.B[,"converged",bb,bb2,nn,3]==TRUE])

resultat.B["MSE",bb,bb2,nn,1] <- mean(resCovar.B[,"bias",bb,bb2,nn,1][resCovar.B[,"converged",bb,bb2,nn,1]==TRUE]**2)

resultat.B["MSE",bb,bb2,nn,2] <- mean(resCovar.B[,"bias",bb,bb2,nn,2][resCovar.B[,"converged",bb,bb2,nn,2]==TRUE]**2)

resultat.B["MSE",bb,bb2,nn,3] <- mean(resCovar.B[,"bias",bb,bb2,nn,3][resCovar.B[,"converged",bb,bb2,nn,3]==TRUE]**2)

resultat.B["prop0.1_OK",bb,bb2,nn,1] <- mypropbis(resCovar.B[,"beta1",bb,bb2,nn,1][resCovar.B[,"converged",bb,bb2,nn,1]==TRUE], Beta1[bb])

resultat.B["prop0.1_OK",bb,bb2,nn,2] <- mypropbis(resCovar.B[,"beta1",bb,bb2,nn,2][resCovar.B[,"converged",bb,bb2,nn,2]==TRUE], Beta1[bb])

resultat.B["prop0.1_OK",bb,bb2,nn,3] <- mypropbis(resCovar.B[,"beta1",bb,bb2,nn,3][resCovar.B[,"converged",bb,bb2,nn,3]==TRUE], Beta1[bb])

resultat.B["prop0.2_OK",bb,bb2,nn,1] <- mypropbis2(resCovar.B[,"beta1",bb,bb2,nn,1][resCovar.B[,"converged",bb,bb2,nn,1]==TRUE], Beta1[bb])

resultat.B["prop0.2_OK",bb,bb2,nn,2] <- mypropbis2(resCovar.B[,"beta1",bb,bb2,nn,2][resCovar.B[,"converged",bb,bb2,nn,2]==TRUE], Beta1[bb])

resultat.B["prop0.2_OK",bb,bb2,nn,3] <- mypropbis2(resCovar.B[,"beta1",bb,bb2,nn,3][resCovar.B[,"converged",bb,bb2,nn,3]==TRUE], Beta1[bb])

resultat.B["power",bb,bb2,nn,1] <- pvalLess.05(resCovar.B[,"pvalue",bb,bb2,nn,1][resCovar.B[,"converged",bb,bb2,nn,1]==TRUE])

resultat.B["power",bb,bb2,nn,2] <- pvalLess.05(resCovar.B[,"pvalue",bb,bb2,nn,2][resCovar.B[,"converged",bb,bb2,nn,2]==TRUE])

resultat.B["power",bb,bb2,nn,3] <- pvalLess.05(resCovar.B[,"pvalue",bb,bb2,nn,3][resCovar.B[,"converged",bb,bb2,nn,3]==TRUE])

}

}

}

######################################################################################################

######################################################################################################

######################################################################################################

##### tableaux de resultats

# Table 1: Bias in b1 in estimating beta1 = 1

res.C <- rbind(resultat.C["mean_bias", 3,1,,], resultat.C["mean_bias", 3,2,,], resultat.C["mean_bias", 3,3,,]) # beta2= 0 / 0.5 / 1

res.B <- rbind(resultat.B["mean_bias", 3,1,,], resultat.B["mean_bias", 3,2,,], resultat.B["mean_bias", 3,3,,]) # beta2= 0 / 0.5 / 1

res <- cbind(res.C, res.B)

round(res, 3)

# Table 2: mean variance in b1 when beta2 = -1

res.C <- rbind(resultat.C["var_bias", 1,3,,], resultat.C["var_bias", 2,3,,], resultat.C["var_bias", 3,3,,])

res.B <- rbind(resultat.B["var_bias", 1,3,,], resultat.B["var_bias", 2,3,,], resultat.B["var_bias", 3,3,,])

res <- cbind(res.C, res.B)

round(res, 2)

# Table 3: proportion with b1 situated between beta1 +/- 0.1 pour beta2 = -1

res.C <- rbind(resultat.C["prop0.1_OK", 1,3,,], resultat.C["prop0.1_OK", 2,3,,], resultat.C["prop0.1_OK", 3,3,,])

res.B <- rbind(resultat.B["prop0.1_OK", 1,3,,], resultat.B["prop0.1_OK", 2,3,,], resultat.B["prop0.1_OK", 3,3,,])

res <- cbind(res.C, res.B)

round(res, 2)

# Table 4: proportion of b1 statistically significant when beta1 = 0 (type 1 error)

res.C <- rbind(resultat.C["power",1,1,,], resultat.C["power",1,2,,], resultat.C["power",1,3,,])

res.B <- rbind(resultat.B["power",1,1,,], resultat.B["power",1,2,,], resultat.B["power",1,3,,])

res <- cbind(res.C, res.B)

round(res, 3)

# Table 5: proportion de b1 statistically significant when beta1 = 0.5 / 1 (power)

res.C <- rbind(resultat.C["power",2,3,,], resultat.C["power",3,3,,])

res.B <- rbind(resultat.B["power",2,3,,], resultat.B["power",3,3,,])

res <- cbind(res.C, res.B)

round(res, 2)

######################################################################################################

######################################################################################################

######################################################################################################

##### Figures 1 à 3

###########################################################################

# Figure 1. Scatter-plots of unadjusted and adjusted estimates of treatment

# effect (b1), for the strong treatment effect (β1=1), a strong continuous

# confounder (β2=-1), and sample size 2×50

###########################################################################

XLIM <- c(-1.1, 3.2)

YLIM <- c(-1.1, 3.2)

x11();

# b1 adjusted on sample vs. b1 unadjusted

par(fig=c(0,1/3,0,1), mar=c(3,3,0.1,0.1), mgp=c(2,0.8,0), cex.axis=1.5, cex.lab=1.2, cex.main=1.2)

plot(resCovar.C[,"beta1",3,3,1,1], resCovar.C[,"beta1",3,3,1,2], xlim = XLIM, ylim = YLIM, main = "", xlab = "Unadjusted", ylab = "Adjusted, sample-based")

abline(h=1, lty=2, col="grey60")

abline(v=1, lty=2, col="grey60")

toto <- loess(resCovar.C[,"beta1",3,3,1,2]~resCovar.C[,"beta1",3,3,1,1], span = 0.2, new=TRUE)

lines(toto$x[order(toto$x)], toto$fitted[order(toto$x)], col="grey60", lwd=3)

# b1 adjusted in true model vs. b1 unadjusted

par(fig=c(1/3,2/3,0,1), mar=c(3,4,0.1,0.1), mgp=c(2,0.8,0), cex.axis=1.5, cex.lab=1.2, cex.main=1.2, new=TRUE)

plot(resCovar.C[,"beta1",3,3,1,1], resCovar.C[,"beta1",3,3,1,3], xlim = XLIM, ylim = YLIM, main = "", xlab = "Unadjusted", ylab = 'Adjusted,true model')

abline(h=1, lty=2, col="grey60")

abline(v=1, lty=2, col="grey60")

toto <- loess(resCovar.C[,"beta1",3,3,1,3]~resCovar.C[,"beta1",3,3,1,1], span = 0.2)

lines(toto$x[order(toto$x)], toto$fitted[order(toto$x)], col="grey60", lwd=3)

# b1 adjusted on sample vs. b1 adjusted in true model

par(fig=c(2/3,1,0,1), mar=c(3,4,0.1,0.1), mgp=c(2,0.8,0), cex.axis=1.5, cex.lab=1.2, cex.main=1.2, new=TRUE)

plot(resCovar.C[,"beta1",3,3,1,3], resCovar.C[,"beta1",3,3,1,2], xlim = XLIM, ylim = YLIM, main = "", xlab = 'Adjusted, true model', ylab = "Adjusted, sample-based")

abline(h=1, lty=2, col="grey60")

abline(v=1, lty=2, col="grey60")

toto <- loess(resCovar.C[,"beta1",3,3,1,2]~resCovar.C[,"beta1",3,3,1,3], span = 0.2)

lines(toto$x[order(toto$x)], toto$fitted[order(toto$x)], col="grey60", lwd=3)

# N=2*50

cor.test(resCovar.C[,"beta1",3,3,1,1], resCovar.C[,"beta1",3,3,1,2])

cor.test(resCovar.C[,"beta1",3,3,1,1], resCovar.C[,"beta1",3,3,1,3])

cor.test(resCovar.C[,"beta1",3,3,1,3], resCovar.C[,"beta1",3,3,1,2])

###############################################################################

# Figure 2. Scatter plots of (sample-based) adjusted estimates of the treatment

# effect (b1) as a function of the estimated confounder effect (b2), with true

# parameter values β1=1 nd β2=-1, for continuous and binary confounders, at

# sample size 2×50. Grey lines represent non-parametric regression functions

# (Lowess)

###############################################################################

XLIM <- c(-4.1, 1.2)

YLIM <- c(-1.1, 3.2)

### continuous covariate (Sample-based : N=2*50, b1=1, b2=-1 (C continu))

x11();

par(fig=c(0,0.5,0,1), mar=c(4,4,2,0), mgp=c(2,0.8,0), cex.axis=1.5, cex.lab=1.2, cex.main=1.2)

plot(resCovar.C[,"beta2",3,3,1,2], resCovar.C[,"beta1",3,3,1,2], xlim = XLIM, ylim = YLIM, main = "", xlab = "", ylab = "")

abline(h=1, lty=2, col="grey60")

abline(v=-1, lty=2, col="grey60")

toto <- loess(resCovar.C[,"beta1",3,3,1,2]~resCovar.C[,"beta2",3,3,1,2], span = 0.2)

lines(toto$x[order(toto$x)], toto$fitted[order(toto$x)], col="grey60", lwd=3)

### binary covariate (Sample-based : N=2*50, b1=1, b2=-1 (C binaire)) !! attention, in 5 simulations b2 did not converge !!

par(fig=c(0.5,1,0,1), mar=c(4,3,2,1), mgp=c(2,0.8,0), cex.axis=1.5, cex.lab=1.2, cex.main=1.2, new=TRUE)

plot(resCovar.B[,"beta2",3,3,1,2][resCovar.B[,"beta2",3,3,1,2] > -17], resCovar.B[,"beta1",3,3,1,2][resCovar.B[,"beta2",3,3,1,2] > -17], xlim = XLIM, ylim = YLIM, main = "", xlab = "", ylab = "")

abline(h=1, lty=2, col="grey60")

abline(v=-1, lty=2, col="grey60")

toto <- loess(resCovar.B[,"beta1",3,3,1,2][resCovar.B[,"beta2",3,3,1,2] > -17]~resCovar.B[,"beta2",3,3,1,2][resCovar.B[,"beta2",3,3,1,2] > -17], span = 0.2)

lines(toto$x[order(toto$x)], toto$fitted[order(toto$x)], col="grey60", lwd=3)

mtext("Treatment effect (b1)" , side=2, outer = TRUE, adj=0.5, padj = 1.25, cex=1.8)

mtext("Confounder effect (b2)", side=1, outer = TRUE, adj=0.52, padj = -1.25, cex=1.8)

mtext("Continuous confounder", side=3, outer = TRUE, adj=0.25, padj = 1.5, cex=1.5)

mtext("Binary confounder" , side=3, outer = TRUE, adj=0.825, padj = 1.5, cex=1.5)

##############################################################################

# Figure 3. Scatter plot of (sample based) adjusted estimates of the treatment

# effect (b1) as a function of the confounder imbalance between the two groups

# (Cohen’s d), with true parameter values β1=1 nd β2=-1, for continuous and

# binary confounders, at sample size 2×50. Grey lines represent non-parametric

# regression functions (Lowess)

##############################################################################

XLIM <- c(-1.2, 1.2)

YLIM <- c(-1.1, 3.2)

### continuous covariate (Sample-based : N=2*50, b1=1, b2=-1 (C continu))

x11();

par(fig=c(0,0.5,0,1), mar=c(4,4,2,0), mgp=c(2,0.8,0), cex.axis=1.5, cex.lab=1.2, cex.main=1.2)

plot(resCovar.C[,"cohen.d",3,3,1,2], resCovar.C[,"beta1",3,3,1,2], xlim = XLIM, ylim = YLIM, main = "", xlab = "", ylab = "")

abline(h=1, lty=2, col="grey60")

abline(v=0, lty=2, col="grey60")

toto <- loess(resCovar.C[,"beta1",3,3,1,2]~resCovar.C[,"cohen.d",3,3,1,2], span = 0.2)

lines(toto$x[order(toto$x)], toto$fitted[order(toto$x)], col="grey60", lwd=3)

### binary covariate (Sample-based : N=2*50, b1=1, b2=-1 (C binaire)) !! attention, in 5 simulations b2 did not converge !!

par(fig=c(0.5,1,0,1), mar=c(4,3,2,1), mgp=c(2,0.8,0), cex.axis=1.5, cex.lab=1.2, cex.main=1.2, new=TRUE)

plot(resCovar.B[,"cohen.d",3,3,1,2][resCovar.B[,"beta2",3,3,1,2] > -17], resCovar.B[,"beta1",3,3,1,2][resCovar.B[,"beta2",3,3,1,2] > -17], xlim = XLIM, ylim = YLIM, main = "", xlab = "", ylab = "")

abline(h=1, lty=2, col="grey60")

abline(v=0, lty=2, col="grey60")

toto <- loess(resCovar.B[,"beta1",3,3,1,2][resCovar.B[,"beta2",3,3,1,2] > -17]~resCovar.B[,"cohen.d",3,3,1,2][resCovar.B[,"beta2",3,3,1,2] > -17], span = 0.2)

lines(toto$x[order(toto$x)], toto$fitted[order(toto$x)], col="grey60", lwd=3)

mtext("Treatment effect (b1)" , side=2, outer = TRUE, adj=0.5, padj = 1.25, cex=1.8)

mtext("Confounder imbalance (Cohen's d)", side=1, outer = TRUE, adj=0.52, padj = -1.25, cex=1.8)

mtext("Continuous confounder", side=3, outer = TRUE, adj=0.25, padj = 1.5, cex=1.5)

mtext("Binary confounder" , side=3, outer = TRUE, adj=0.825, padj = 1.5, cex=1.5)
